# Will the European Union reach the United Nations Millennium declaration target of a 50% reduction of tuberculosis mortality between 1990 and 2015?

**DOI:** 10.1186/s12889-017-4544-9

**Published:** 2017-07-06

**Authors:** Marieke J. van der Werf, Sandro Bonfigli, Frantiska Hruba

**Affiliations:** European Centre for Disease Prevention and Control, Granitsväg 8, 17165 Solna, Stockholm Sweden

**Keywords:** Tuberculosis, Death, Millennium development goal, European Union

## Abstract

**Background:**

The Millennium Development Goals (MDG) provide targets for 2015. MDG 6 includes a target to reduce the tuberculosis (TB) death rate by 50% compared with 1990. We aimed to assess whether this target was reached by the European Union (EU) and European Economic Area countries.

**Methods:**

We used Eurostat causes of death data to assess whether the target was reached in the EU. We calculated the reduction in reported and adjusted death rates and the annual average percentage decline based on the available data.

**Results:**

Between 1999 and 2014, the TB death rate decreased by 50%, the adjusted death rate by 56% and the annual average percentage decline was 5.43% (95% confidence interval 4.94–6.74) for the EU. Twenty of 26 countries reporting >5 TB deaths in the first reporting year reached the target of 50% reduction in adjusted death rate.

**Conclusions:**

The EU reached the MDG target of a 50% reduction of the TB death rate and also the annual average percentage decline was larger than the 2.73% needed to reach the target. The World Health Organization ‘End TB Strategy’ requires a further reduction of the number of TB deaths of 35% by 2020 compared to 2015, which will challenge TB prevention and care services in the EU.

## Background

In 2000, the heads of state adopted the United Nations Millennium Declaration [1]. The road map towards the implementation of this Declaration outlines potential strategies for action that are designed to meet the goals and commitments made in the Millennium Declaration and provides 8 goals for 2015 [2]. One of the eight goals, goal 6, specifically focusses on infectious diseases, i.e. human immunodeficiency virus (HIV)/acquired immunodeficiency syndrome (AIDS), malaria and other diseases. Within this goal there is a target “to have halted by 2015 and begun to reverse the incidence of malaria and other major diseases”. For tuberculosis (TB), the indicators for this target are prevalence and death rates associated with TB and proportion of tuberculosis cases detected and cured under directly observed treatment short course. In 1991, the World Health Assembly (WHA) set targets to detect at least 70% of incident cases and to successfully treat at least 85% of TB patients by 2000 [3]. The indicators for prevalence and death rate have been translated into targets for 2015 by the Stop TB Partnership, i.e. reduce prevalence and death rates by 50%, compared with their levels in 1990 [4].

Annually, the World Health Organization (WHO) assesses whether the world is on track for reaching the set targets [5]. The Millennium Development Goal (MDG) target for the TB incidence rate i.e. a declining incidence by 2015 has been met globally. The target for TB mortality will most likely not be achieved by the end of 2015. Also the TB prevalence target will not be achieved globally.

In the European Union and European Economic Area (EU/EEA), the TB notification rates, used as a proxy for TB incidence, have been declining since the start of surveillance of TB in 1995, i.e. from 23 per 100,000 in 1995 to 13 per 100,000 in 2014 [6, 7]. In the EU/EEA there is no direct information on TB prevalence since TB prevalence cannot be directly measured in the EU/EEA due to the excessively large samples sizes that would be needed to come to a reliable TB prevalence estimate [8]. Therefore, information on TB prevalence is derived from modelling studies. The modelling suggested that the TB prevalence target will not be achieved for the WHO European Region [5], however, no modelling results are available for the EU. The modelling also suggested that the 50% reduction in TB mortality rate will not be achieved in the WHO European Region. Whether the EU/EEA will reach a 50% reduction in TB mortality has not been assessed.

In the EU/EEA, substantial human and financial resources are available to fight diseases compared to other regions in the world. Also, the area has suffered a less substantial increase in HIV and subsequent increase in TB as seen in other areas. Thus, the EU/EEA is well placed to reach the MDG and Stop TB Partnership targets. We therefore set out to analyse available TB mortality data for the EU/EEA to assess whether the target set for TB mortality was reached.

## Methods

We obtained data from the Eurostat database (http://ec.europa.eu/eurostat/data/database) on 19 April 2017 for all EU and EEA countries and for the total of the EU (28 countries). For France we used the data reported for France including overseas territories. The following data were available: Population data on the 1st of January for all EU and EEA countries by age and sex for 1994 to 2016; Deaths for 1994 to 2015; Cause of death, All causes of death (International Classification of Disease [ICD] 10 codes A00-Y89) excluding ICD-10 codes S00-T98 for 1994 to 2014 (last available year); Cause of death, ill-defined codes, Symptoms, signs and abnormal clinical and laboratory findings, not elsewhere classified (ICD-10 codes R00-R99) for 1994 to 2014 (last available year); and Cause of death, tuberculosis (ICD-10 codes A15-A19_B90). Cause of death data refer to the underlying cause which - according to the WHO definition - is “the disease or injury which initiated the train of morbid events leading directly to death, or the circumstances of the accident or violence which produced the fatal injury”. Mortality from TB is defined as the number of deaths caused by TB in HIV-negative people. TB deaths among HIV-positive people are classified as HIV deaths in ICD-10. Until 2010, the causes of death data were collected on voluntary basis from EU and EEA Member States. From 2011 onwards reporting of causes of death is required by a regulation [9]. The Eurostat webpage provides information on the quality of the data, especially on accuracy, comparability and coherence.

We calculated the TB death rate from the number of TB deaths reported in the causes of death data registration and the population data on the 1st of January in the same year. Thereafter, we calculated the change in TB death rate from the TB death rate in the first year with available data (1994 or later) and the TB death rate in 2014 (last year with data available).

We also assessed the change in death rate for the adjusted TB death rate. The adjusted TB death rate takes into account incomplete coverage (deaths with no cause documented) and ill-defined causes of death (ICD-10 codes R00–R99) and assumes non-differential misclassification. We adjusted the TB death rate as described in the technical appendix of the Global Report 2014 [10]. The number of death without an assigned cause of death was determined by taking the difference between “Deaths” (total number of deaths) and “All causes of death” (ICD-10 codes A00-Y89, total number of death with a cause of death). It was assumed that the proportion of TB deaths among deaths without an allocated cause of death was the same as the proportion of TB deaths among deaths with a Cause of death reported. Ill-defined Cause of death codes (ICD-10 codes R00-R99) were redistributed to TB death by assuming that the proportion of deaths attributable to TB was the same in the ill-defined Cause of death as in the defined Cause of deaths. Thus, the adjusted number of TB deaths was calculated as follows, *dc* (1−*g*), where *d* is the number of TB deaths obtained from the cause of death registration, *c* denotes coverage (i.e. the number of deaths with a documented cause of death divided by the total number of deaths) and *g* denotes the proportion of ill-defined causes of death.

A > 50% decline in death rates translates in an average annual percentage decline of 2.73% (1-[1–0.5] ^ [1/25]). We assessed whether this average annual decline was reached by calculating the average annual percentage decline in death rates from the adjusted TB death rate using a log-linear univariate regression model with Stata software, version 14.0.

For countries reporting up to 5 TB deaths in the first year with data available we do not provide the change in death rate and the annual average percentage decline in death rates.

## Results

For 21 of the 28 EU Member States and for Iceland and Norway causes of death data were available for 1994 from Eurostat (Table 1). One EU Member State (Bulgaria) started reporting causes of death data in 1995, two EU Member States (Latvia and Slovakia) in 1996, two (Croatia and Romania) in 1999 and Cyprus first reported for 2004. All EU Member States reported causes of death data for 2014 and Norway (Table 2). The most recent causes of death data reported by Iceland were from 2009. Liechtenstein never reported causes of death data to Eurostat. For the EU (28 countries) causes of death data were available from 1999 onwards.

**Table 1:** Tuberculosis (TB) death rate per 100,000 population in first year with available data in European Union and European Economic Areas Member States

Member State	First year causes of death data available	Nr TB deaths in first year with causes of death data available	Population number in first year with causes of death data available	TB death rate per 100,000 population in first year with causes of death data available
Austria	1994	113	7,928,746	1.43
Belgium	1994	138	10,100,631	1.37
Bulgaria	1995	332	8,427,418	3.94
Croatia	1999	214	4,527,459	4.73
Cyprus	2004	3	722,893	0.41
Czech Republic	1994	132	10,334,013	1.28
Denmark	1994	44	5,196,642	0.85
Estonia	1994	143	1,476,952	9.68
Finland	1994	114	5,077,912	2.25
France	2001	1061	60,979,315	1.74
Germany	1994	1014	81,338,093	1.25
Greece	1994	107	10,489,871	1.02
Hungary	1994	653	10,350,010	6.31
Iceland^a^	1994	4	265,064	1.51
Ireland	1994	52	3,583,154	1.45
Italy	1994	733	56,842,392	1.29
Latvia	1996	289	2,469,531	11.70
Liechtenstein^a^	-	-	30,310	-
Lithuania	1994	413	3,671,296	11.25
Luxembourg	1994	5	400,200	1.25
Malta	1994	1	373,161	0.27
Netherlands	1994	140	15,341,553	0.91
Norway^a^	1994	71	4,324,815	1.64
Poland	1994	1416	38,504,707	3.68
Portugal	1994	337	9,974,391	3.38
Romania	1999	2152	22,488,595	9.57
Slovakia	1996	77	5,367,790	1.43
Slovenia	1994	29	1,989,408	1.46
Spain	1994	754	39,458,489	1.91
Sweden	1994	103	8,745,109	1.18
United Kingdom	1994	571	57,788,017	0.99
European Union (28 countries)	1999	8780	486,577,953	1.80

^a^European Economic Area Member States and not included in the European Union total

**Table 2:** Tuberculosis (TB) death rate per 100,000 population in 2014 in European Union and European Economic Areas Member States and change in death rate compared to first year with causes of death data available

Member State	Nr TB deaths in 2014^a^	Population number in 2014^a^	TB death rate, per 100,000 population 2014	First year causes of death data available	TB death rate per 100,000 population in first year with causes of death data available	Reduction in death rate between first year with cause of death data available and 2014 (%)
Austria	70	8,506,889	0.82	1994	1.43	42
Belgium	40	11,180,840	0.36	1994	1.37	74
Bulgaria	126	7,245,677	1.74	1995	3.94	56
Croatia	40	4,246,809	0.94	1999	4.73	80
Cyprus	4	858,000	0.47	2004	0.41	-^c^
Czech Republic	42	10,512,419	0.40	1994	1.28	69
Denmark	12	5,627,235	0.21	1994	0.85	75
Estonia	28	1,315,819	2.13	1994	9.68	78
Finland	40	5,451,270	0.73	1994	2.25	67
France	449	65,942,093	0.68	2001	1.74	61
Germany	315	80,767,463	0.39	1994	1.25	69
Greece	52	10,926,807	0.48	1994	1.02	53
Hungary	87	9,877,365	0.88	1994	6.31	86
Iceland^b^	7	319,368	2.19	1994	1.51	-^c^
Ireland	24	4,605,501	0.52	1994	1.45	64
Italy	288	60,782,668	0.47	1994	1.29	63
Latvia	66	2,001,468	3.30	1996	11.7	72
Liechtenstein^b^	-	37,129	-	-	-	-
Lithuania	225	2,943,472	7.64	1994	11.25	32
Luxembourg	1	549,680	0.18	1994	1.25	-^c^
Malta	1	425,384	0.24	1994	0.27	-^c^
Netherlands	35	16,829,289	0.21	1994	0.91	77
Norway^b^	12	5,107,970	0.23	1994	1.64	86
Poland	534	38,017,856	1.40	1994	3.68	62
Portugal	206	10,427,301	1.98	1994	3.38	42
Romania	1125	19,947,311	5.64	1999	9.57	41
Slovakia	34	5,415,949	0.63	1996	1.43	56
Slovenia	21	2,061,085	1.02	1994	1.46	30
Spain	279	46,512,199	0.60	1994	1.91	69
Sweden	30	9,644,864	0.31	1994	1.18	74
United Kingdom	346	64,351,155	0.54	1994	0.99	46
European Union (28 countries)	4520	506,973,868	0.89	1999	1.80	50

^a^Iceland 2009

^b^European Economic Area Member States and not included in the European Union (28 countries)

^c^Not calculated because country reported 5 or less TB deaths in first year with TB death data available

The TB death rate per 100,000 population ranged between 0.27 and 11.7 for the first year with available death data in the different EU/EEA countries. For the EU (28 countries) the death rate was 1.81 per 100,000 in 1999. In the last year with causes of death data available the death rate ranged from 0.18 to 7.64 per 100,000, and was 0.89 for the EU (28 countries).

The death rate decreased by 50% between 1999 and 2014 in the EU (28 countries), Table 2. A decrease in death rate of more than 75% was observed for Croatia, Denmark, Estonia, Hungary the Netherlands, and Norway. Twenty countries reached the target of 50% reduction in mortality rate in the period with data available. For the 22 EU countries with data available in 1994 and in 2014 the death rate decreased by 64%, i.e. from 1.85 in 1994 to 0.66 in 2014.

Adjusting the death rate for incomplete coverage of the causes of death registration and ill-defined causes of death changed the country specific TB death rates slightly (Table 3). For 23 countries adjusting the death rate resulted in a change of less than 5% in the first year with causes of death data available and for 22 countries the change in the death rate for the year 2014 (Iceland 2009) was less than 5%. For the EU (28 countries) total the changes were more substantial. The death rate changed from 1.80 to 2.09 (15.5%) when adjusted in 1999 and from 0.89 to 0.92 (3.4%) in 2014. The changes in 1999 were due to a coverage of the cause of death registration of only 89.2% and a proportion of ill-defined causes of death of 3.2% and the changes in 2014 were mainly due to a proportion of ill-defined causes of death of 3.5%. The coverage was 100% in 2014. The adjustments resulted in a decrease in death rate of 56% between 1999 and 2014 in the EU (28 countries), Table 3.

**Table 3:** Adjusted TB death rates taking into account incomplete coverage (deaths with no cause documented) and ill-defined causes of death for 1994 or first year with data available and 2014 and the change in death rate

Member State	Adjusted TB death rate per 100,000 population for first year with data available	Adjusted TB death rate, per 100,000 population 2014^a^	Reduction in death rate between first year with cause of death data available and 2014 (%)	Annual average percentage decline (95% confidence interval)
Austria	1.44	0.84	42	3.45 (1.86–5.01)
Belgium	1.42	0.38	74	5.67 (4.82–6.52)
Bulgaria	4.12	1.79	57	4.24 (3.34–5.13)
Croatia	4.87	0.94	81	9.13 (7.30–10.92)
Cyprus	0.47	0.46	-^c^	-^c^
Czech Republic	1.28	0.40	69	5.62 (4.11–7.11)
Denmark	0.92	0.23	75	6.75 (5.51–7.97)
Estonia	10.07	2.17	78	8.50 (7.35–9.64)
Finland	2.26	0.74	67	6.63 (5.64–7.62)
France	1.86	0.75	60	6.39 (5.81–6.97)
Germany	1.28	0.40	69	5.44 (4.69–6.19)
Greece	1.10	0.51	54	2.03 (0.09–3.14)
Hungary	6.32	0.88	86	9.27 (8.72–10.34)
Iceland^b^	1.52	2.21	-^c^	-^c^
Ireland	1.46	0.52	64	4.59 (3.29–5.88)
Italy	1.31	0.48	63	4.65 (4.00–5.30)
Latvia	12.32	3.37	73	8.42 (7.09–9.74)
Liechtenstein^b^	-	-	-	-
Lithuania	11.62	7.79	33	2.48 (1.67–3.28)
Luxembourg	1.33	0.19	-^c^	-^c^
Malta	0.27	0.23	-^c^	-^c^
Netherlands	0.95	0.22	77	7.35 (6.53–8.16)
Norway^b^	1.71	0.25	85	7.53 (6.32–8.73)
Poland	4.00	1.51	62	4.63 (4.18–5.09)
Portugal	3.80	2.10	45	4.58 (3.86–5.30)
Romania	9.58	5.71	40	4.14 (3.30–4.98)
Slovakia	1.44	0.64	56	5.46 (3.58–7.31)
Slovenia	1.51	1.03	32	3.54 (1.46–5.57)
Spain	1.95	0.61	69	5.83 (5.35–6.31)
Sweden	1.20	0.32	73	6.57 (5.37–7.76)
United Kingdom	1.00	0.55	45	3.34 (2.88–3.80)
European Union (28 countries)	2.09	0.92	56	5.43 (4.94–6.74)

^a^Iceland 2009

^b^European Economic Area Member States and not included in the European Union (28 countries)

^c^Not calculated because country reported 5 or less TB deaths in first year with TB death data available

The annual average percentage decline in death rates was 5.43% (95% confidence interval 4.94–6.74) for the EU (28 countries), Table 3. Two countries, Greece and Lithuania, did not reach the 2.73% target for the average annual percentage decline. It is likely that in two additional countries, Austria and Slovenia, this target was not reached as well. All average decline estimates were statistically highly significant.

## Discussion

The Millennium Development Declaration target of 50% reduction in TB death rate was reached by the EU (28 countries) in 2014 compared to the first year with TB death data available for the EU (28 countries).

Comparing the decline in TB death rates in the EU with those in other areas shows that rates are comparable. However, the decline was more modest in the EU compared to the decline reported in the United States and Cuba. In the United States, the age-adjusted TB mortality rate declined from 2.22 per 100,000 person-years in 1990 to 0.47 per 100,000 person-years in 2006, i.e. a decline of 79% [11]. TB mortality in Cuba declined from 0.4 per 100,000 population in 1998 to 0.2 in 2007 [12]. TB mortality rates in Brazil seemed to be higher i.e. 5.9 per 100,000 in 1980 and 3.1 per 100,000 in 2001 [13]. Differences in the quality of the health care system, public health interventions and the socio economic situation of the population can explain the observed differences in TB death rates and their decline.

Six EU/EEA countries did not reach the target of a 50% reduction in death rate within the period of observation, i.e. up to 2014. They reported between 144 and 15,906 TB cases in 2014 [7]. The TB surveillance data do not show a uniform epidemiological pattern that can explain why they did not reach the target. Three countries showed a decrease in TB notification rates between 1995 and 2014; three showed an initial increase in TB notification rates followed by a decrease [14]. In Austria, Lithuania, and Portugal the TB treatment outcomes were less favourable than the average of the EU/EEA and in Romania, Slovenia, and the United Kingdom more favourable. With respect to drug resistance, the percentage of cases with multidrug-resistant TB differed considerably, from 0% in Slovenia to 21.5% in Lithuania and showed an increasing trend in Austria, a decreasing and then increasing trend in the United Kingdom, was stable in Lithuania, Portugal and Slovenia, and increased and thereafter decreased in Romania.

The TB mortality rate will likely further decline if TB cases are diagnosed early [15, 16]. Currently not all TB cases are diagnosed promptly in the EU as is shown by several studies [17, 18]. This may be due to insufficient knowledge of patients and health care workers about TB in areas with a low TB incidence [19]. Poor performing health systems may diagnose TB late with as a consequence a higher case fatality rate in diagnosed TB cases. In addition, a significant percentage of TB case may be diagnosed after death. In San Francisco, 4% of all TB cases were diagnosed after death between 1986 through 1995 [20]. In an earlier period, 1985–1988, 5.1% of the TB cases reported in United States were diagnosed at death [21]. Studies from other areas, such as Taiwan, show similar percentages [22]. Thus, a further reduction of the TB mortality rate will require investments in raising awareness of TB and early diagnosis.

To prevent mortality from TB, identified cases need to receive prompt and adequate treatment. Knowledge of health care workers about tuberculosis treatment is often insufficient [23]. This may be an explanation for the fact that the TB treatment success rate observed in the EU/EEA has not reached the target of successfully treating at least 85% of the TB cases [3, 7]. Eight percent of the TB cases diagnosed in 2013 died during treatment [7]. Those who die during TB treatment may die of TB, i.e. ICD-10 codes A15-A19 and B90, and be counted as TB death in the causes of death registration, however they may also die of other causes [24, 25]. In 2014, 4039 TB cases died during TB treatment [26]. In the same year, 4532 TB deaths were registered in the vital registration database of Eurostat. Not all deaths due to TB occur during TB treatment, also after TB treatment there seems to be increased mortality [27] which may be due to remaining sequelae [28, 29]. Adequate treatment and support to ensure treatment adherence may reduce TB mortality, both during TB treatment and after TB treatment [30, 31].

Our analysis has a few limitations, related to the available data. We used data reported by EU/EEA Member States to Eurostat. TB death data were not reported by all Member States for all years and they were only available from 1999 onwards for the EU (28 countries) and not from 1990, the baseline year for measurement of the targets. This may be due to the fact that Commission Regulation No 328/2011 on Community statistics on public health and health and safety at work, as regards statistics on causes of death only became effective on 5 April 2011 [9].

By comparing the first year with causes of death information available, 1999, with the last year with causes of death data available (2014), we have made a conservative estimate of the decline in TB death rates since the MDG target is for a reduction in TB death rate between 1990 and 2015. Also, there is no evidence for an increase in TB death rates between 1990 and 1999. The total TB death rate in the EU in countries with data available for the years 1994 and 1999 decreased in this period.

The quality of vital registration systems can be measured using several indicators, e.g. coverage and completeness, and percentage of ill-defined conditions (ICD–10 codes R00–R99) [32]. More recently the vital statistics performance index was suggested [33]. Most EU/EEA Member States, except for Italy and Greece, had a high vital statistics performance index suggesting that the quality of the data is sufficient to draw conclusions.

A global assessment shows that the world will not reach a 50% reduction in mortality rate by 2015 compared with 1990 [5]. This assessment is mainly based on indirect estimates, using case fatality ratios and estimates of TB incidence, since vital registration cause of death data are not widely available. The strengths of our analysis is that it is based on direct data on causes of death reported by countries to Eurostat.

In 2016, the implementation of the World Health Organization ‘End TB Strategy’ has started [34, 35]. This strategy contains ambitious targets, including a target for a 35% reduction in tuberculosis deaths by 2020 and a 95% reduction by 2035 (compared with 2015). For the EU/EEA this results in a death rate of approximately 0.58 and 0.04 per 100,000 population respectively. Reaching this will be challenging for TB prevention and care services in the EU/EEA and will need a whole of government approach [36].

The MDGs are succeeded by the sustainable development goals. In its summit on 25 September 2015 in New York, the General Assembly of the United Nations adopted the 2030 Agenda for Sustainable Development. This agenda includes a target on ending several epidemics including the tuberculosis epidemic by 2030 [37]. It remains to be seen what indicators will be used to measure this. However, there will be a need for reliable surveillance and vital registration systems to measure progress towards this target.

## Conclusion

Although the EU/EEA has reached the MDG target of a 50% reduction in death rates, new strategies and targets will need continues efforts and solid systems that can provide the data to measure progress towards the targets.

## Abbreviations

AIDS: Acquired immunodeficiency syndrome
EEA: European economic area
EU: European Union
HIV: Human immunodeficiency virus
ICD: International classification of disease
MDG: Millennium development goal
TB: Tuberculosis
WHA: World Health Assembly
WHO: World Health Organization

## References

[1] United Nations General Assembly. United Nations Millennium Declaration, Resolution Adopted by the General Assembly, 18 September 2000, A/RES/55/2. 2000. http://www.un.org/millennium/declaration/ares552e.htm.

[2] United Nations. Road map towards the implementation of the United Nations Millennium Declaration: Report of the Secretary-General; 2001. http://www.un.org/documents/ga/docs/56/a56326.pdf.

[3] World Health Assymbly (1991). Resolution WHA. 44.8. Forty-fourth world health assembly, Geneva, 6–16 may, 1991. Resolutions and decisions.

[4] Stop TB Partnership. The global plan to stop TB, 2006–2015. Geneva: World Health Organization. p. 2006.

[5] World Health Organization (2015). Global tuberculosis report 2015.

[6] Antoine D, Schwoebel V, Veen J, Raviglione MC, Rieder HL (1998). Surveillance of tuberculosis in the WHO European Region 1995-1996. Euro Surveill.

[7] European Centre for Disease Prevention and Control, WHO Regional Office for Europe (2016). Tuberculosis surveillance and monitoring in Europe, 2016.

[8] Glaziou P, van der Werf MJ, Onozaki I, Dye C, Borgdorff MW, Chiang CY (2008). Tuberculosis prevalence surveys: rationale and cost. Int J Tuberc Lung Dis.

[9] Commission regulation (EU) of 5 April 2011, implementing Regulation (EC) No 1338/2008 of the European Parliament and of the Council on Community statistics on public health and health and safety at work, as regards statistics on causes of death. Official Journal of the European Union. 2008;L354:70–81. http://eur-lex.europa.eu/LexUriServ/LexUriServ.do?uri=OJ:L:2008:354:0070:0081:EN:PDF.

[10] World Health Organization (2014). Global tuberculosis report 2014.

[11] Jung RS, Bennion JR, Sorvillo F, Bellomy A (2010). Trends in tuberculosis mortality in the United States, 1990-2006: a population-based case-control study. Public Health Rep.

[12] Gonzalez E, Risco GE, Borroto S, Perna A, Armas L (2009). Tuberculosis mortality trends in cuba, 1998 to 2007. MEDICC Rev.

[13] Hino P, da Costa-Junior ML, Sassaki CM, Oliveira MF, Villa TC, dos Santos CB (2007). Time series of tuberculosis mortality in Brazil (1980-2001). Rev Lat Am Enfermagem.

[14] European Centre for Disease Prevention and Control Surveillance Atlas of Infectious Diseases. [Internet] [cited 2016 February]; Available from: https://ecdc.europa.eu/en/surveillance-atlas-infectious-diseases

[15] Lui G, Wong RY, Li F, Lee MK, Lai RW, Li TC (2014). High mortality in adults hospitalized for active tuberculosis in a low HIV prevalence setting. PLoS One.

[16] Greenaway C, Menzies D, Fanning A, Grewal R, Yuan L, FitzGerald JM (2002). Delay in diagnosis among hospitalized patients with active tuberculosis--predictors and outcomes. Am J Respir Crit Care Med.

[17] Cruz-Ferro E, Ursua-Diaz MI, Taboada-Rodriguez JA, Hervada-Vidal X, Anibarro L, Tunez V (2014). Epidemiology of tuberculosis in Galicia, Spain, 16 years after the launch of the Galician tuberculosis programme. Int J Tuberc Lung Dis.

[18] Saldana L, Abid M, McCarthy N, Hunter N, Inglis R, Anders K (2013). Factors affecting delay in initiation of treatment of tuberculosis in the Thames Valley, UK. Public Health.

[19] Jurcev Savicevic A, Popovic-Grle S, Milovac S, Ivcevic I, Vukasovic M, Viali V (2008). Tuberculosis knowledge among patients in out-patient settings in split, Croatia. Int J Tuberc Lung Dis.

[20] DeRiemer K, Rudoy I, Schecter GF, Hopewell PC, Daley CL (1999). The epidemiology of tuberculosis diagnosed after death in San Francisco, 1986-1995. Int J Tuberc Lung Dis.

[21] Rieder HL, Kelly GD, Bloch AB, Cauthen GM, Snider DE (1991). Tuberculosis diagnosed at death in the United States. Chest.

[22] Wu YC, Lo HY, Yang SL, Chou P (2014). Factors correlated with tuberculosis reported after death. Int J Tuberc Lung Dis.

[23] van der Werf MJ, Langendam MW, Huitric E, Manissero D (2012). Knowledge of tuberculosis-treatment prescription of health workers: a systematic review. Eur Respir J.

[24] Walpola HC, Siskind V, Patel AM, Konstantinos A, Derhy P (2003). Tuberculosis-related deaths in Queensland, Australia, 1989-1998: characteristics and risk factors. Int J Tuberc Lung Dis.

[25] Takahara M (2004). Clinical evaluation of causes of death in patients with pulmonary tuberculosis. Kekkaku.

[26] European Centre for Disease Prevention and Control, WHO Regional Office for Europe (2017). Tuberculosis surveillance and monitoring in Europe, 2017.

[27] Miller TL, Wilson FA, Pang JW, Beavers S, Hoger S, Sharnprapai S (2015). Mortality hazard and survival after tuberculosis treatment. Am J Public Health.

[28] Pasipanodya JG, Miller TL, Vecino M, Munguia G, Garmon R, Bae S (2007). Pulmonary impairment after tuberculosis. Chest.

[29] Hnizdo E, Singh T, Churchyard G (2000). Chronic pulmonary function impairment caused by initial and recurrent pulmonary tuberculosis following treatment. Thorax.

[30] Pablos-Mendez A, Sterling TR, Frieden TR (1996). The relationship between delayed or incomplete treatment and all-cause mortality in patients with tuberculosis. JAMA.

[31] Kayigamba FR, Bakker MI, Mugisha V, De Naeyer L, Gasana M, Cobelens F (2013). Adherence to tuberculosis treatment, sputum smear conversion and mortality: a retrospective cohort study in 48 Rwandan clinics. PLoS One.

[32] Mathers CD, Fat DM, Inoue M, Rao C, Lopez AD (2005). Counting the dead and what they died from: an assessment of the global status of cause of death data. Bull World Health Organ.

[33] Phillips DE, Lozano R, Naghavi M, Atkinson C, Gonzalez-Medina D, Mikkelsen L (2014). A composite metric for assessing data on mortality and causes of death: the vital statistics performance index. Popul Health Metr.

[34] World Health Organization Sixty-Seventh World Health Assembly (2014). A67/11. Global strategy and targets for tuberculosis prevention, care and control after 2015.

[35] Uplekar M, Weil D, Lonnroth K, Jaramillo E, Lienhardt C, Dias HM (2015). WHO’s new end TB strategy. Lancet.

[36] Lonnroth K, Migliori GB, Abubakar I, D'Ambrosio L, de Vries G, Diel R (2015). Towards tuberculosis elimination: an action framework for low-incidence countries. Eur Respir J.

[37] United Nations Sustainable Development Knowledge Platform. [Internet] [cited 2015 October]; Available from: https://sustainabledevelopment.un.org/?menu=1300

